# Attitude and knowledge towards clozapine among outpatients prescribed clozapine in Durban, KwaZulu-Natal

**DOI:** 10.4102/sajpsychiatry.v24i0.1316

**Published:** 2018-09-20

**Authors:** Saeeda Paruk, Shawkat A Roojee

**Affiliations:** 1 Discipline of Psychiatry, School of Clinical Medicine, University of KwaZulu-Natal, South Africa

## Abstract

**Background:**

Increased patient satisfaction towards their treatment is associated with improved adherence. Poor adherence to antipsychotics is predictive of relapse and eventual need for hospitalisation. There is very limited literature on patients’ knowledge and perception of antipsychotic – or more specifically clozapine – treatment.

**Aim:**

To describe the perception and knowledge of clozapine amongst patients prescribed clozapine at two psychiatric units in Durban, KwaZulu-Natal.

**Methods:**

A cross-sectional survey of 94 adult patients receiving clozapine was conducted using a socio-demographic questionnaire, chart review and the Drug Attitude Inventory scale (DAI-30), an indirect measure of adherence.

**Results:**

The participants were predominantly male (*n* = 63, 67.0%) and black people (*n* = 71, 75.5%). The most common diagnosis was schizophrenia (*n* = 58, 61.7%), and majority surveyed received clozapine for more than one year (*n* = 93, 98.9%). Ninety-one (96.8%) participants knew the names of medications, and 71 (75.5%) participants were aware of the common side effects of clozapine. Eighty-six (91.5%) patients were aware of the need for white blood cell testing and 50 (53.2%) patients reported being informed by their mental health practitioner about the risk of gaining weight with 41 (43.6%) patients complaining of this side effect.

The total number of correct responses to 30 questions on the DAI-30 (*N* = 2457) compared favourably to the incorrect responses (*N* = 363). Eighty-seven patients were found to be adherent, and 7 patients were considered non-adherent as per DAI total scores.

Significant associations were between negative DAI score suggesting non-adherence and patient educational level (*p* = 0.001), past history of defaulting medication (*p* = 0.001), medication efficacy perception (*p* = 0.01) and being able to discuss concerns with doctor (*p* = 0.02), compared to those with a positive score on DAI.

**Conclusion:**

A high rate of self-reported positive attitude and knowledge towards clozapine was found. The findings highlight the critical role of psycho-education and therapeutic alliance in attitude towards medication and hence adherence.

